# Magnetic nanostructures functionalized with a derived lysine coating applied to simultaneously remove heavy metal pollutants from environmental systems

**DOI:** 10.1080/14686996.2020.1865114

**Published:** 2021-01-22

**Authors:** Olivija Plohl, Marjana Simonič, Ken Kolar, Sašo Gyergyek, Lidija Fras Zemljič

**Affiliations:** a Faculty of Mechanical Engineering, Laboratory for Characterization and Processing of Polymers, University of Maribor, Maribor, Slovenia; b Faculty of Chemistry and Chemical Engineering, University of Maribor, Maribor, Slovenia; c Department for Materials Synthesis, Jožef Stefan Institute, Ljubljana, Slovenia

**Keywords:** Advanced nanotechnology application, nanostructured functional surface, derived lysine coating, hybrid magnetic nanoadsorbent, heavy metal removal, environmental systems contamination, 102 Porous / Nanoporous / Nanostructured materials, 103 Composites, 212 Surface and interfaces, 301 Chemical syntheses / processing, 501 Chemical analyses, Adsorption, environmental applications

## Abstract

The pollution of environmental systems with heavy metals is becoming a serious problem worldwide. These contaminants are one of the main issues in sludge (which is considered waste) and can even have harmful effects if the sludge is not treated properly. Thus, the development of a novel functional magnetic nanoadsorbent based on a derived lysine is reported here, which can be efficiently applied for metal removal from sludge. Magnetic nanoparticles were coated with silica layer and further modified by covalent bonding of derived lysine. The morphology of the nanomaterial, its nano-size and the silica layer thickness were analyzed by transmission electron microscopy. The successful silanization of the lysine derivative to the silica-coated magnetic nanostructures was investigated by several physicochemical characterization techniques, while the magnetic properties were measured with a vibrating sample magnetometer. The synthesized nanostructures were used as adsorbents for simultaneous removal of most critical heavy metals (Cr, Zn, Cu) from real complex sludge suspensions. The main practical adsorption parameters, pH of the native stabilized sludge, adsorbent amount, time, and adsorbent regeneration were investigated. The results show promising adsorption properties among currently available adsorbents (the total equilibrium adsorption capacity was 24.5 mg/g) from the sludge with satisfactory nanoadsorbent reusability and its rapid removal. The stability of the nanoadsorbent in the sludge, an important but often neglected practical parameter for efficient removal, was verified. This work opens up new possibilities for the development of high-quality magnetic nanoadsorbents for metal pollutants applied in various complicated environmental fields and enables waste recovery.

## Graphical abstract

**Figure:**
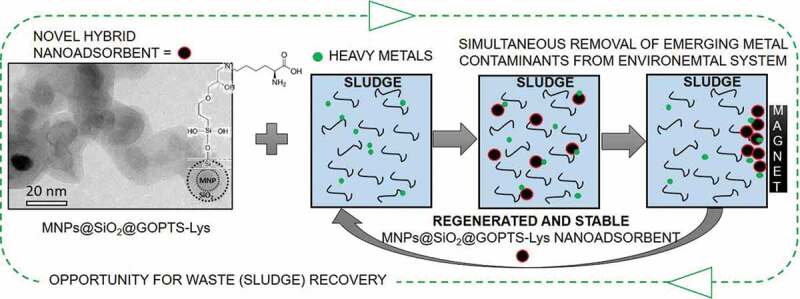

## 1. Introduction

At present, industrialization and urbanization have led to the accumulation of large quantities of toxic metals in environmental systems, which has become a major environmental treatment problem [1–3] requiring urgent solutions. In particular, environmental systems such as sludge from the wastewater treatment process tend to concentrate heavy metals due to the physico-chemical processes involved in treatment. Remarkably, it has been estimated that sludge production in the EU reached 13 million tons of dry matter by 2020. In fact, this is a growing problem worldwide, as sludge production continues to increase enormously with population growth, while quality standards and EU regulations are becoming more stringent [1,4]. As traditional disposal routes are no longer recommended, sludge managers are faced with the challenge of finding cost-effective and innovative solutions while responding to environmental, regulatory, and public pressures [5–8]. Therefore, the elimination of toxic metals from environmental systems such as sludge has become a high priority. Otherwise, sludge is rich in nutrients such as nitrogen and phosphorus and also contains valuable organic matter, which makes this type of waste suitable for application on land as a potential fertilizer or organic soil improver [2,9].

Although the heavy metal contamination of sludge depends on the location and type of sludge produced, the main heavy metal contaminants are chromium, zinc, and copper [10,11]. This is due to the fact that these metals are widely used in various branches of the processing industry, which has led to their discharge and enormous accumulation in the sludge [12,13]. In addition, they can bio-accumulate in plants and be deposited in the human body through the food chain, and the excessive amount of metals can lead to serious dangerous health problems [13–15]. Accordingly, it is paramount importance to remove the excess of hazardous heavy metals from problematic sludges in order to allow recycling into products with high nutrient values. This would make it possible to use the treated sludge for agricultural applications without adverse effects on human health.

A variety of physical and chemical methods such as ion exchange, coagulation/flocculation, adsorption, chemical oxidation, ozone treatment, and membrane filtration have been tried to remove these pollutants from wastewater [13,16–18]. However, many of these conventional removal techniques have some limitations. They can cause serious secondary pollution, the processes are complex, uneconomical, and produce large quantities of sludge that can only be treated with great difficulty [13]. Contrary to the current state-of-the-art, adsorption allows for flexibility in adsorbent design, higher efficiency, lower operating costs together with lower energy consumption and process reversibility, where the same adsorbents can be used several times [18,19]. Various materials such as nanomaterials, graphene oxide, zeolite, polymeric materials, and activated carbon have been used for toxic metal removal [13,18,19]. However, there are still some disadvantages, such as low adsorption capacity, long adsorption time, but the main disadvantage is the complicated and costly removal of the used adsorbent after the adsorption process (filtration, centrifugation), which is not economically attractive [13,18,19]. It should be noted that only a few studies on the removal of heavy metals from the real environmental systems have been carried out with different adsorbents [20–25], whereas there is only one report on heavy metal removal from real sludge suspension using chitosan-based magnetic NPs [4]. Other studies mainly focus only on metal removal from river water [20] or (industrial) wastewater [21,22] using magnetic nanoparticles, and the on use of nZVI-based adsorbents for metal removal from industrial metal-rich wastewater streams [23–25]. It is therefore of utmost importance to develop a stable and efficient nanoadsorbent that can be used directly for complex sludge contaminated with heavy metals.

In this respect, nanoscale magnetic adsorbents may appear to be the right solution for a highly efficient adsorption process. This can be correlated with the magnetic response of the nanoadsorbent, which serves as a cheap and simple separation process. Furthermore, a large specific surface area allows more active adsorption sites for the removal of heavy metals. Promising magnetic nanoadsorbents are based on magnetic iron oxide (MNPs), as they do not pose an environmental hazard and are economically attractive [16,26]. However, it has been shown that the bare MNP surface is relatively inert [27], and these types of MNPs do not appear to be very efficient in the adsorption process [22,28–30]. The density of the -OH groups and the surface reactivity of MNPs can be improved with a thick layer of amorphous silica. This allows further chemical bonding to additional substrates with specific functionality, such as various derived organosilanes [31]. In addition, silica is porous and increases the surface area, it is environmentally friendly, chemically stable, and affordable from an economic point of view [32].

Recently, it has been shown that amino acids are good chelating agents for metal removal from aqueous environments. The functionalization of amino acids on a good solid surface is strongly recommended to achieve high efficiency together with stability [33]. In fact, amino acids contain some reactive groups such as carboxylate and amine groups, which are able to form stable chelates with various heavy metals and so far these biomolecules have been little researched in the field of heavy metal remediation [34]. Some publications pointed out that different amino acids have already been used to modify various types of adsorbents (e.g. Fe_3_O_4_-(GO-MWCNTs) hybrid magnetic composites, graphene oxide, crosslinked chitosan resin, clay materials, cellulose adsorbent, etc.) to remove metal contaminants [28,33,35–41]. For example, Fe_3_O_4_ NPs modified with arginine and lysine were synthesized and showed promising efficiency in the removal of arsenic from model water system. The arsenic adsorption capacities of arginine and lysine-based MNPs were 29.14 and 23.86 mg/g, respectively, which was twice as high as that of bare MNPs (12.12 mg/g), illustrating out the importance of surface modification with functional groups [28]. However, the use of e.g. GO adsorbent-based materials is limited by the low dispersibility in water, the low porosity and the tendency to aggregate in water, which consequently leads to a lower adsorption efficiency. Moreover, the main disadvantage of using non-magnetic-based adsorbents is the difficult and expensive separation process for its large-scale application. Polyacrylonitrile fibers covalently modified with lysine were synthesized for removal of U(VI) from artificial seawater [42]. Nevertheless, the high costs involved correlated to adsorbent removal and the inconvenience of industrialization hinder the selection of an optimal choice. Indeed, an important but often overlooked fact is that many reports using lysine-based adsorbent [28,33,35–41] focus only on single metal removal from model solutions mostly with not permanently bonded lysine and do not investigate adsorption in real and complex systems, which is crucial for commercial purposes.

Although to the best of our knowledge there have been some publications on the production of lysine-based adsorbents, the adsorption of problematic metals (i.e. Zn, Cu, and Cr according to Sewage Sludge Directive 86/278/EEC [10]) from a real sludge suspension using magnetic-silica nanomaterials with a core-shell structure, further modified with functional derived lysine coating has not been reported so far.

This paper presents a study of epoxy-organosilane modified lysine and its functionalization on MNPs@SiO_2_ for the simultaneous removal of heavy metals from real environmental systems. The engineered nanoadsorbent and its synthesis parameters were investigated in detail. The exhaustive characterization revealed a successful chemical binding of derived lysine to core-shell MNPs@SiO_2_. The effect of adsorbent dosage, contact time, and regeneration of the novel nanoadsorbent was investigated in a real sludge suspension for the most critical metals. Finally, an important factor in the real application is the strong and permanent binding of the adsorbent to the nanocarrier, which was verified by X-ray photoelectron spectroscopy (XPS) and electrokinetic analysis.

## 2. Experimental section

### 2.1. Chemicals and reagents

Details about used chemicals and reagents can be found in Supplemental Material (SM).

### 2.2. MNPs preparation and adsorption of citric acid (MNPs@CA)

Maghemite-based MNPs were synthesized by coprecipitation under air atmosphere. Details of the synthesis are given elsewhere [43]. In short, an aqueous Fe^2+^ and Fe^3+^ solution (V = 500 mL) with the ratio *n*(Fe^2+^): *n*(Fe^3+^) = 2.4 : 1 was prepared using ferrous sulfate reagent salts and ultrapure water. For iron hydroxides precipitation, dilute aqueous ammonia solution was slowly added to iron salt solution at pH 3. Afterwards, 250 mL aqueous ammonia solution (25%) was quickly added to the above mixture and the black slurry was stirred for another 30 min. The MNPs were then washed several times with prepared dilute aqueous ammonia solution and with ultrapure water. To produce a stable colloidal MNPs dispersion, the adsorption of citric acid (CA) according to reference [43] was used. Synthesized bare MNPs (1.2 g) were redispersed in 60 mL MiliQ water. The CA aqueous solution was added to the MNPs dispersion (5 mL of 0.5 g mL^−1^). The pH value of the resulting dispersion was adjusted to 5.2 with diluted aqueous ammonia solution and allowed to stand for 1.5 h at 80°C under reflux. Finally, the pH of the cooled MNPs@CA dispersion was increased to pH~10 using aqueous ammonia solution (25%).

### 2.3. SiO^2^-coated MNPs (MNPs@SiO^2^)

The coating of the colloidal dispersion of MNPs@CA with a silica shell (MNPs@SiO_2_) was done in a similar way as reported in ref [31], with some modifications. First, NH_4_OH (25%) was added to MNPs@CA dispersion (15 mg mL^–1^) to achieve a pH = 10.6. Secondly, the mixture was stirred for 15 minutes and then added to the solution of TEOS and EtOH at a concentration of 10 mg mL^−1^. Using 25% NH_4_OH the pH was adjusted to pH = 10.6. Then, the coating process was left for 2 h under constant stirring. The successfully produced core-shell MNPs@SiO_2_ were washed with absolute EtOH and MiliQ water.

### 2.4. Chemical derivatization of GOPTS and lysine (GOPTS-Lys) and its silanization onto MNPs@SiO^2^ (i.e. MNPs@SiO^2^@GOPTS-Lys)

Amino acid lysine was initially derived with an organic epoxy functional reactive group to ensure a permanent and strong covalent bond to the magnetically based nanocarrier. Derived lysine with organosilane thus produced was silanized under controlled conditions to MNPs covered with the silica layer with high density of reactive silanol groups, which improves the density coverage of the adsorption ligand [44]. This is then reflected in the formation of permanently bound derived lysine on the magnetic carrier, which should result in highly efficient metal removal, especially in more complex environmental suspensions such as sludge.

In order to carry out the synthesis systematically, some parameters were taken into account, such as the amount of derived lysine, which can influence the dispersion stability of MNPs@SiO_2_ during the silanization process, thus leading to a lower coating efficiency. First, the estimated MNPs@SiO_2_ specific surface area was 95 m^2^ g^−1^ [43] and the surface density of the Si-OH groups was approximately 5 groups per 1 nm^2^ [32]. Secondly, it was assumed that the one organosilane epoxy group from GOPTS reacts with the nucleophilic primary amino group from the lysine backbone. Finally, it was predicted that each Si-OH group reacts with a methoxy group from derived GOPTS-Lys.

**Scheme 1.**
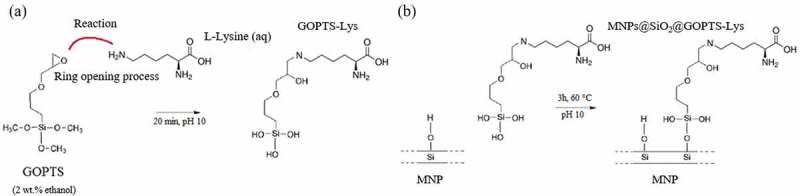
Derivatization process of GOPTS and Lys (a) and proposed chemical linkage of GOPTS-Lys to MNPs@SiO2 (b)

To prepare 100 mg MNPs@SiO_2_@GOPTS-Lys, the estimated amount of Lys (five monomers Lys per 1 nm^2^ MNPs@SiO_2_) was dissolved in 20 mL MiliQ water. The pH of the alkaline Lys solution was lowered to pH = 10 with 0.1 M HCl. At the same time, epoxy-containing organosilane GOPTS was dissolved in EtOH (2 wt.% solution, the amount of GOPTS corresponding to about 5 molecules/1 nm^2^ MNPs@SiO_2_). In order to achieve chemical binding (as suggested in Scheme 1), the prepared solution of GOPTS was slowly added to an aqueous Lys solution, while keeping the pH constant at 10. The need for alkaline conditions under which the primary amine nucleophiles react successfully with the epoxy group has already been pointed out [44]. The transparent and clear solution thus prepared was stirred for 20 minutes on a magnetic stirrer. Afterwards, chemically crosslinked GOPTS-Lys was added to an alkaline aqueous MNPs@SiO_2_ dispersion (pH = 10) with a particle concentration of 4 mg mL^−1^. At this pH according to electrokinetic measurements, the repulsive negative forces due to negatively charged silanol groups among MNPs@SiO_2_ allow a stable dispersion during the silanization process. Subsequently, the dispersion was refluxed for silanization of GOPTS-Lys to MNPs@SiO_2_ for 3 h at 60°C. After completion of the silanization process the hot dispersion was cooled down. Then, the MNPs@SiO_2_@GOPTS-Lys were magnetically separated from the dispersion and cleaned several times with moderately acidic MiliQ water (pH 4) to remove unreacted reagents. The proposed derivatization mechanism is schematically shown in Scheme 1a, while the silanization process from GOPTS-Lys to MNPs@SiO_2_ is schematically shown in Scheme 1b.

### 2.5. Methods: characterization of the nanoadsorbent

#### 2.5.1. Structural X-ray powder diffraction

X-ray powder diffraction (XRD) was used to check the purity of the as-synthesized MNPs, MNPs@SiO_2_ and MNPs@SiO_2_@GOPTS-Lys (which were used in the form of the dried product) and the crystal structure. The instrument used for the measurement was a PANalytical X` Pert PRO (Malvern Panalytical, Almelo, Netherlands) with CuKα radiation *λ*_CuKα_ = 1.5406 Å and with the following parameters of the measurements: 2-Theta range was chosen from 25 to 70° with a scan rate of 0.385° min^−1^ and 2 s per step.

#### 2.5.2. Transmission electron microscopy

The morphology of nanostructures was investigated with transmission electron microscopy (TEM; Jeol JEM−2100, Tokyo, Japan). For TEM the investigations, the nanomaterials were deposited on a copper-grid-supported, perforated, transparent carbon film. The equivalent diameter of the nanomaterials, the thickness of the silica shell and the specific surface area were estimated from different TEM images using Gatan Digital Micrograph software and calculated accordingly. EDS spectra were acquired using the Oxford Instruments ISIS 300 EDS spectrometer (JED 2300 EDS; Jeol, Tokyo, Japan).

#### 2.5.3. Surface chemistry: ATR-FTIR Spectroscopy and XPS analysis

The attenuated total reflection Fourier transform infrared (ATR-FTIR) spectra were recorded with a PerkinElmer Spectrum GX NIR FT-Raman spectrometer (Waltham, Massachusetts, USA). The ATR accessories contained a diamond crystal. To perform the measurements, dry samples were placed on a diamond crystal and pressed into the thick layer. All spectra including background were obtained in the wave number range of 400–4000 cm^−1^ at room temperature with a resolution of 2 cm^−1^. Finally, all recorded spectra were deconvoluted with baseline correction and with smoothing filter. The survey and high-resolution C 1s, N 1s, Si 2p, and Fe 2p spectra were obtained with an XPS instrument PHI-TFA 5600 XPS spectrometer from Physical Electronics Inc. (Chanhassen, Minnesota, USA). The base pressure in the XPS analysis chamber was about 6 × 10^−8^ Pa. The samples were excited by X-rays with a monochromatic Al Kα1.2 radiation (1486.6 eV) operated at 200 W. The photoelectrons were detected by a hemispherical analyzer positioned at a 45° angle to the normal to the sample surface. The energy resolution was about 0.6 eV. Spectra were taken from at least two locations on each sample, using the radius of the analyzed area of 400 μm. An additional electron gun was used to compensate for the possible charging effect of the samples. The surface element concentrations were calculated from the spectra of the survey scans using the Multipak software, version 9.6.0. A C 1s high-resolution spectrum was employed at a binding energy of 284.8 eV to correct the binding energy scale.

#### 2.5.4. Electrokinetic measurements of zeta potential

Electrokinetic measurements by the electrophoresis technique (DLS ZetaSizer Nano ZS, Malvern Instruments Ltd, Malvern, UK) were used to monitor the zeta potential of prepared dispersions in aqueous media. To perform the zeta potential measurements, folded disposable capillary cells with electrodes were used, which were filled up with 0.1 wt% prepared dispersion and placed in a sample holder. Here the respective pH value for the measurement was adjusted accordingly with either 0.1 M HCl or 0.1 M NaOH at a constant temperature of 25°C in 1 mM NaCl background. After the measurement was completed, the data were recorded using the manufacturer’s Malvern Zetasizer software version 7.12.

#### 2.5.5. Charge and pK determination with potenciometric titration

Potentiometric titration of pure and derived lysine was performed for the determination of both total charge and *p*K values. Using 0.1 M KOH and 0.1 M HCl aqueous solutions as titrant, the pH-dependent titration was carried out at 1.5< pH <11.5. The combined glass electrode (Mettler Toledo InLab Reach 225) was equipped with a double burette instrument (Mettler T-70). All solutions for potentiometric titrations were prepared with ultrapure water with a very low carbonate content (<10^–6^ M). The ionic strength of the lysine solution was adjusted to 0.1 M with 3 M KCl before starting the titration. The HCl-KOH blank titration was carried out under the same conditions as mentioned above. Further details on the charge calculations and the *p*K value determination can be found elsewhere [45].

#### 2.5.6. Thermogravimetric analysis

A thermogravimetric analysis (TGA) was performed to determine the weight fraction of the mass loss. The measurement was performed on the dried powdery sample (approx. 5 mg) at a heating rate of 10 K min^−1^ using a Mettler TGA thermogravimetric analyzer (TGA/SDTA 851 Mettler Toledo, Switzerland). The samples to be analyzed were placed in 60 μL aluminum oxide crucibles and heated in an oxygen atmosphere from 25°C to 600°C.

#### 2.5.7. Magnetic properties

The saturation magnetizations and room-temperature magnetization curves of different samples were characterized using a vibrating-sample magnetometer (VSM, Lake Shore 7307, Westerville, Ohio, USA). For sample preparation, the nanoparticle powder was placed in a plastic holder and sealed. The measurements were performed at 300 K with an external magnetic field in the range of –10^4^ to 10^4^ Oe using a magnetic separator (Model L-1, S.G. Frantz Co., Tullytown, Pennsylvania, USA).

### 2.6. Heavy metal adsorption from complex sludge suspension

The industrial sewage sludge formed as a by-product from wastewater treatment plant was supplied from a municipal wastewater treatment plant in Maribor, Slovenia. The typical sludge composition and present inorganic and organic species are explained in [11,46]. When the various drying experiments were carried out, it was found that it contains about 2 wt.% dry matter and the pH of the latter was 10.7. The batch adsorption experiments were carried out in 50 mL flasks in 20 mL sludge with 2 wt.% of dry matter under vigorous magnetic stirring, overcoming the binding of nanoadsorbent to the wall or to the magnetic stirrer in native sludge pH (alkaline). The amount of nanoadsorbent (2.5, 5, 10, 20, 35 mg) was added to the flask together with the 20 mL 2 wt.% sludge at constant temperature (298 K). The effect of the contact time was investigated at an adsorption contact time of 5 to 120 min with an optimal dosage of the nanoadsorbent of 0.5 mg mL^−1^ (10 mg), whereas in other batch experiments the optimal contact time was set to 2 h. After the adsorption process, the magnetic nanoadsorbent was immediately removed with usage of permanent magnet. The supernatant and its amount of the most problematic remaining heavy metals (i.e. Zn, Cu, Cr) according to the Slovenian legislation (Official Gazette the Republic of Slovenia, No. 99/13 in 56/15), which were also indicated as the most critical metals in the Sewage Sludge Directive 86/278/EEC, were determined by atomic absorption spectroscopy (AAS) following standard method SIST ISO 11047 [47]. Prior AAS the samples were digested by nitric and hydrofluoric acid in microwave. Standard solution, containing Zn, Cu and Cr were prepared in concentration range 0.1 mg L^−1^ up to 2.0 mg L^−1^. The measurements were performed at 213.9 nm for Zn, at 324.7 nm for Cu and at 357.9 nm for Cr. Measurement uncertainty was determined at 10% for all enumerated metals. The removal efficiency and the adsorption capacity were calculated with the following expressions written in Eq. 1 and Eq. 2:

(1) $$Removal\;efficiency\;\left(\%\right)\;=\;(c_{o}-c_{t})/c_{o}\times \;100\%$$

(2) $$\begin{array}{r} Adsorption\;capacity\;(mg\;g^{-1})\\ \;\;\;\;\;\;\;\;=\;(c_{o}-c_{t})\;\times m_{dm}/m \end{array}$$

where *c*_o_ is the initial metal content in sludge (mg/kg of dry matter) and the *c_t_* is the metal concentration in sludge (mg/kg of dry matter) after adsorption treatment, *m* is the mass of the nanoadsorbent (g) and the *m*_dm_ is the mass of the sludge dry matter used for experiment (kg).

**Scheme 2.**
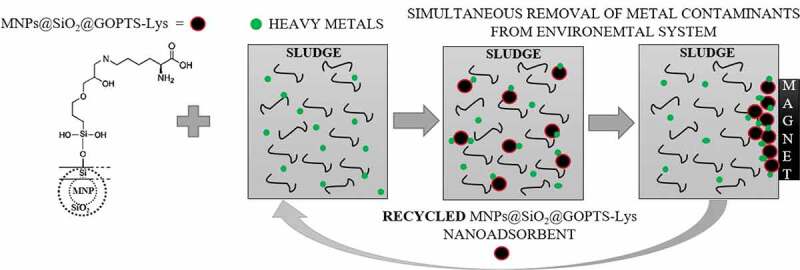
Overall concept for the use of the nanoadsorbent MNPs@SiO2@GOPTS-Lys in the simultaneous removal of heavy metals from polluted and complex environmental system as sludge, including its recyclability

Recycling investigations were carried out with the adsorption batch experiment similar to the above at optimal conditions (pH = 10.7, contact time 2 h, γ(MNPs@SiO_2_@GOPTS-Lys in sludge) = 1 mg mL^–1^). After adsorption, the nanoadsorbents were collected on a permanent magnet, cleaned with MiliQ water, and dried. They were then immersed for desorption studies in 10 mL of 0.1 mol L^−1^ Na_2_EDTA (where EDTA stands for ethylenediaminetetraacetic acid), which has been shown to be the most efficient with the highest desorption capacity among many eluents [48], and desorption was performed for 2 h. Afterwards, MNPs@SiO_2_@GOPTS-Lys were collected again, washed with MiliQ water, dried, and used again for the regeneration experiment in the next cycle. All experiments were conducted in triplicate and the results were reported as an average value with a confidence level of 95%. The overall concept for the use of the nanoadsorbent MNPs@SiO_2_@GOPTS-Lys in the simultaneous removal of heavy metals from polluted and complex environmental systems such as sludge, including their recyclability, is shown in Scheme 2.

## 3. Results and discussion

### 3.1. Effect of lysine linkage and nanoadsorbent characterization

**Figure 1.**
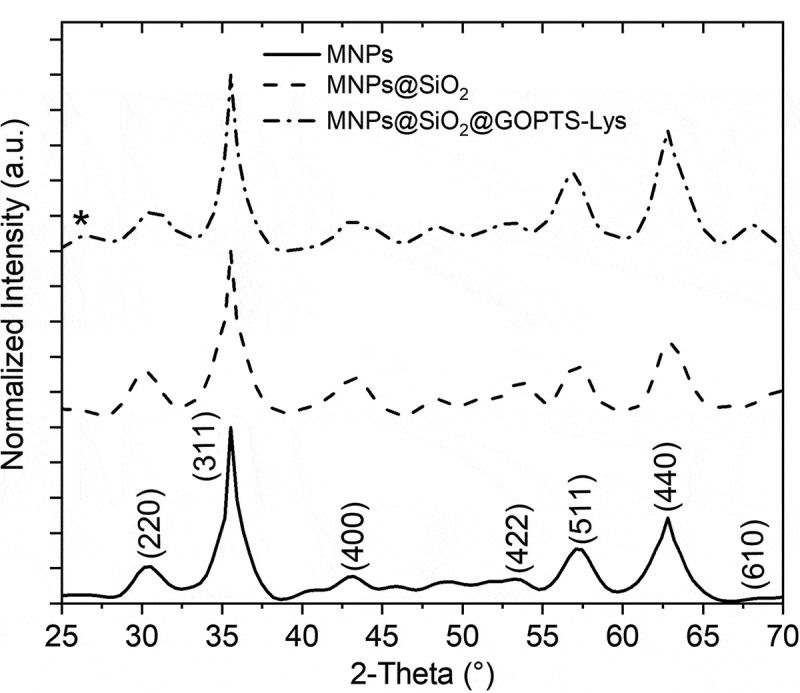
XRD patterns of MNPs with different surface modification steps. Asterix marks the broad hump typical for amorphous materials such as amorphous SiO_2._

**Figure 2.**
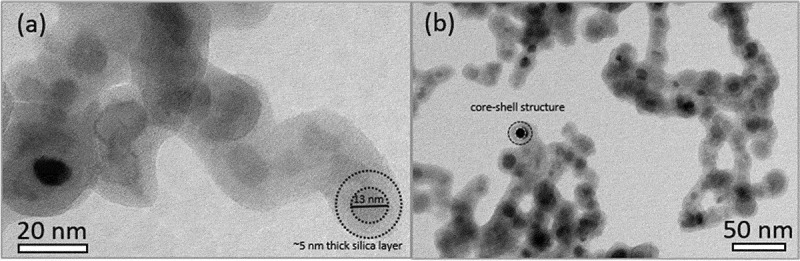
TEM images of MNPs@SiO_2_ (a) and MNPs@SiO_2_@GOPTS-Lys (b) nanostructures

The crystal structure of the bare MNPs and other surface-modified magnetic nanoparticles was verified by XRD analysis (Figure 1), and the crystal structure was assigned to the maghemite independently of various modification processes. The diffraction peaks corresponded to the standard reference card typical for the crystal structure of the maghemite with cubic spinel crystal structure (JCPDS 39–1346, cubic space group P4_1_32). The broad hump at about 25–28° for spectra of MNPs@SiO_2_ and MNPs@SiO_2_@GOPTS-Lys is a characteristic of amorphous materials such as silica [49], and has been attributed to amorphous SiO_2_. Furthermore, the broadening of the diffraction peaks indicates the nano-crystallinity of the synthesized MNPs-based nanoparticles. A further surface modification using an amorphous silica layer and the derivative lysine did not cause any changes in the magnetic maghemite crystal structure. The morphology and nanoscale before and after functionalization of MNPs@SiO_2_ with GOPTS-Lys was investigated with TEM (Figure 2). The MNPs are visible as core of the nanostructure and have a size of ~13 nm. The amorphous silica layer with ~5 nm thickness is uniformly deposited around the MNPs in the form of a core-shell structure (Figure 2(a,b)). The amorphous silica layer is visible as areas of lighter contrast, surrounding the darker spherical MNPs (Figure 2). Careful experimental work on the formation of the silica layer on MNPs confirmed that a silica layer through a process of heterogeneous nucleation surrounded each of the MNPs. To investigate the effect of derivative lysine on the morphology of MNPs@SiO_2_, TEM images of MNPs@SiO_2_@GOPTS-Lys were also taken. No obvious influence on the MNPs@SiO_2_ morphology was observed when an additional silanization step was applied (Figure 2(b)). From this, it can be concluded that GOPTS-Lys had no influence on the morphology of the silica coating. The energy-dispersive X-ray spectroscopy (EDS) analysis of MNPs@SiO_2_@GOPTS-Lys (see Figure S1 in SM) revealed the presence of all constituents except N, which is well below the detection limit [50].

**Figure 3.**
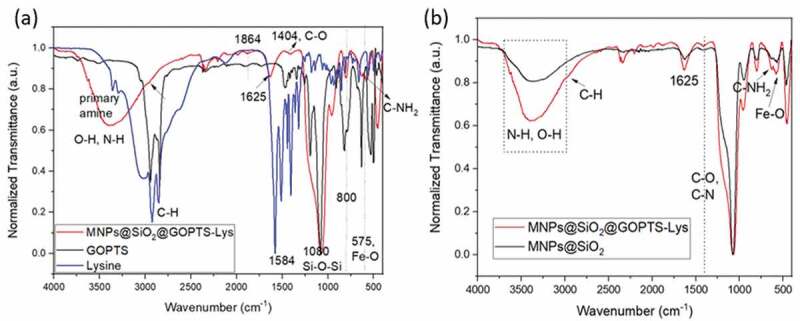
Comparison of FTIR spectra for MNPs@SiO_2_@GOPTS-Lys, pure lysine and GOPTS (a), and for MNPs@SiO_2_ and MNPs@SiO_2_@GOPTS-Lys (b)

The analysis of the surface infrared spectroscopy confirmed the successful formation of both layers; silica and lysine onto MNPs (Figure 3). The band at 575 cm^−1^ (Figure 3) corresponds to the Fe-O vibrations of Fe_2_O_3_ [27] and confirms the presence of the MNPs. The band at 1080 cm^−1^ indicates the presence of an SiO_2_ coating at MNPs@SiO_2_ and is assigned to the asymmetric Si-O-Si stretching vibrations [51]. Also at 950 cm^−1^ for Si-OH stretching, 800 cm^−1^ for Si-O-Si stretching, and 463 cm^−1^ for Si-O-Si bending indicates the presence of an amorphous silica layer. The lysine FTIR spectrum (Figure 3(a)) shows typical signals between 1605 and 1555 cm^−1^ corresponding to asymmetric CO_2_ stretching and symmetrical CO_2_ stretching at 1424 and 1393 cm^−1^. The zwitterionic state of lysine was confirmed with signals between 3100 and 2600 cm^−1^, which are typical for NH_3_ vibrations, antisymmetric deformation at 1665 to 1585 cm^−1^, symmetric deformation at 1530 and 1490 cm^−1^. The C-H stretching vibrations can be seen at 3300 to 2500 cm^−1^. Signals at 3358 and 3288 cm^−1^ represent the N-H bond of primary amines, which is not charged [36,52,53]. The peaks at 2932 and 2845 cm^−1^, which are attributed to the asymmetric stretching of CH_2_ and the symmetrical stretching of the CH groups, are seen, but with lower intensity at MNPs@SiO_2_@GOPTS-Lys, indicating the presence of lysine. After the derivatization reaction of GOPTS with lysine and its silanization on MNPs@SiO_2_, the bands corresponding to the epoxy vibration [54] (at 1270 and 830 cm^−1^) are no longer clearly visible and decrease significantly (Figure 3(a)). The latter agrees to a large extend with the proposed chemical linking mechanism (Scheme 1). Especially, the band at 627 cm^−1^, which corresponds to the C-NH_2_ vibrations, the band at 1404 cm^−1^, which is attributed to the C-O and C-N groups from Lys [54,55] and the less intensive C-H stretching at 2932 and 2845 cm^−1^ suggest a functionalization of GOPTS-Lys on MNPs@SiO_2_(Figure 3(b)).

**Figure 4.**
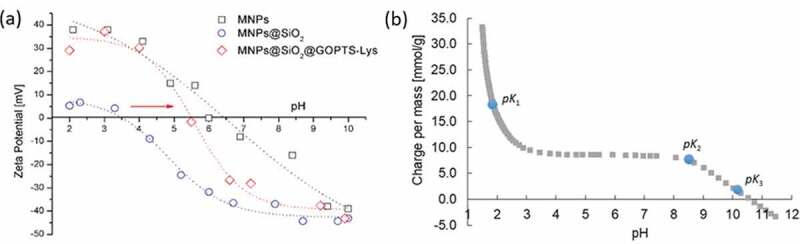
MNPs, MNPs@SiO_2_ and MNPs@SiO_2_@ GOPTS-Lys zeta potential measurements over a wide pH range (a); pH-dependent potentiometric titration for pure lysine (b)

Various surface modification steps of MNPs with silica layer and further with GOPTS Lys derivative were monitored with zeta potential (ZP) and are shown in Figure 4(a). The bare MNPs show an isoelectric point (IEP) at pH = 6.5, which corresponds to the maghemite IEP known from the literature [27]. In contrast to the MNPs, the silica-coated MNPs exhibit negative ZP values over almost the entire pH range due to the presence of negatively charged silanol groups [29], with the IEP at pH ~3.5. Obviously, silanization of GOPTS-Lys to MNPs@SiO_2_ shifts the IEP is shifted to higher (pH_IEP_ ≈ 5.5), an alkaline pH range with more positive ZP values (Figure 4(a)). The shift of the IEP from MNPs@SiO_2_@GOPTS-Lys to a higher pH value indicates on the successful binding of GOPTS-Lys to MNPs@SiO_2_. At a pH below the IEP the amino groups on the surface of MNPs@SiO_2_@GOPTS-Lys were protonated and exhibit positive charge (of lysine origin) [28,56,57]. However, some negative charge may also occur due to negatively charged derived lysine carboxylic groups as well as to silanol groups from the silica coating if not all silanol groups from the silica coating have been covalently modified and the derived lysine layer was not homogeneous. Comparing the ZP measurement with the potentiometric titration of pure lysine (Figure 4(b)), a partial covering of MNPs@SiO_2_ with derived lysine can be determined. It must also be considered that the primary amino group of lysine is not accessible because it is crosslinked with organosilane.

The potentiometric titration curve showed that the pure lysine protonates/deprotonates in the plateaus at pH = 1 and pH = 10.7 in 3-*p*K form with a positive net charge in this range (Figure 4(b)). Since Lys is chemically linked to the GOPTS and silanized to MNPs@SiO_2_ (shown in Scheme 1), the pH_IEP_ ≈ 5.5 is close to the *pK*_2_ of pure lysine, indicating a successful chemical coupling. Additionally, this also supports the fact that pure lysine is in a nearly deprotonated state, which expresses its nucleophilicity when crosslinked to GOPTS at pH 10, while the *pK*_3_ for the second primary amine group is at pH 10. After derivatization of the pure lysine primary end-amino group with GOPTS (Scheme 1), the amount of primary amines was reduced (due to the decrease of positive charge per mass) (see Figure S2 in SM). From this, the derivatization yield of about 25% was determined. Furthermore, the first *pK_1_* was shifted from 1.9 to 2.7 due to the changed protonation behavior of the primary amines, the polymer structure and the milieu of the dissociable groups were changed.

**Figure 5.**
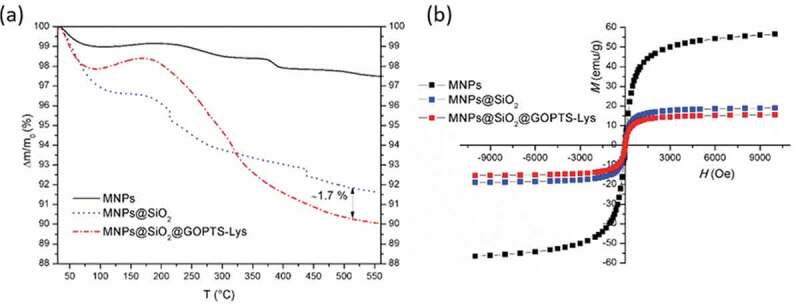
Mass loss as a function of temperature for bare MNPs, MNPs@SiO_2_ and MNPs@SiO_2_@GOPTS-Lys (a). Magnetization curves (at room temperature) for bare MNPs, MNPs@SiO_2_ and MNPs@SiO_2_@GOPTS-Lys (b)

The mass loss as a function of temperature was measured with TGA (Figure 5(a)). Bare MNPs show a small mass loss (2.5 wt%), which is most likely correlated with the physisorbed water and/or solvent residues. A greater loss of mass was measured for silica-coated MNPs (6 wt.%), and the latter may be due to a higher content of silanol groups. Two main weight loss steps were observed in the TGA curve for MNPs@SiO_2_@GOPTS-Lys. The first weight loss step was observed in the temperature range 30–150°C, which is due to the mass loss caused by the -OH release. The second weight loss step in the temperature range could be attributed to the decomposition of the derived lysine [28]. The amount of derived lysine on MNPs@SiO_2_ was determined to be 1.7%. From the mass loss in relation to GOPTS-Lys, a rough estimate of the Lys-based ligand density can be determined [58]. The ligand density together with the mass loss accounts to be 1 molecule GOPTS-Lys per nm^2^. From this and the fact that the mass losses were small, it can be assumed that the surface of MNPs@SiO_2_ was most likely not completely covered. If we consider the proposed chemical coupling mechanism (Scheme 1), 1 amino group and 1 carboxyl group could be used for metal ion chelation in alkaline medium at 1 nm^2^ of MNPs@SiO_2_@GOPTS-Lys.

The room-temperature magnetization curves for bare MNPs, silica-coated MNPs and MNPs@SiO_2_@GOPTS-Lys are presented in Figure 5(b). For bare MNPs the saturation magnetization was determined to be 56.5 emu/g. After coating with the ~5 nm thick SiO_2_ layer (Figure 2), the saturation magnetization of bare MNPs decreased from 56.5 emu/g to 18.0 emu/g. It is generally known the dependence of magnetic properties on the shape, size, and the amount of the magnetic phase [27]. Therefore, in magnetic nanocomposites, the non-magnetic phase, such as the silica layer and further modified with a derived lysine layer, reduces the saturation magnetization. After silanization of MNPs@SiO_2_ with the GOPTS-Lys, it was found that the saturation magnetization of the latter decreased to 16.5 emu/g due to the presence of an additional non-magnetic material. In fact, the modification of MNPs@SiO_2_ with a derived amino acid had no significant effect on the saturation magnetization and had a sufficiently high magnetic force [30,59,60] which could be easily separated from the sludge suspension with a permanent magnet. The absence of coercivity and remanence for bare and differently modified MNPs point to the superparamagnetic properties for all nanomaterials.

### 3.2. The results of real sludge treatment

#### 3.2.1. Effect of nanoadsorbent amount on the simultaneous removal of metals from sludge

**Figure 6.**
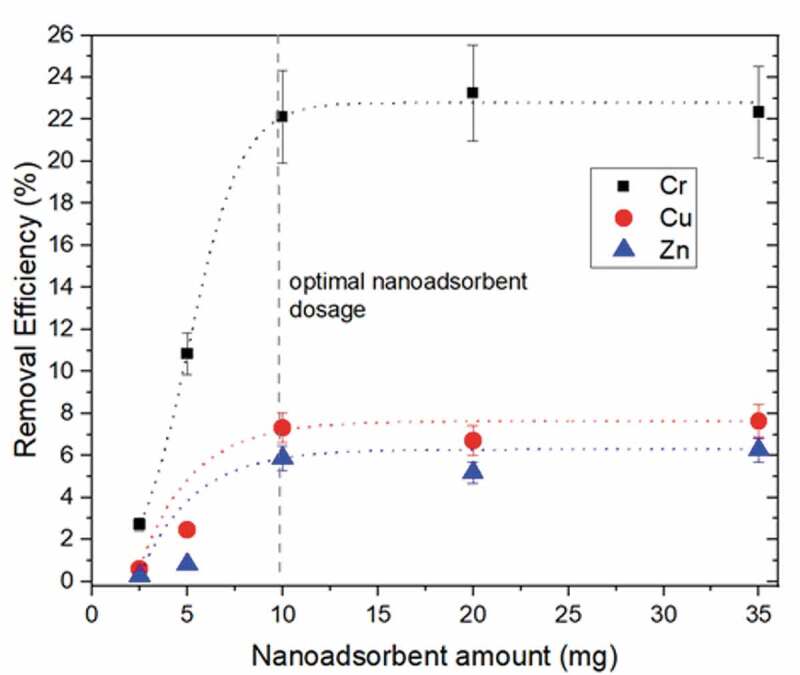
Effect of the MNPs@SiO_2_@GOPTS-Lys nanoadsorbent amount on removal efficiency of Cr, Cu and Zn from sludge suspension at pH = 10.7, adsorption contact time was 2 h, 2 wt.% sludge suspension at T = 298 K

The effect of the nanoadsorbent quantity was investigated in the range of 2.5–35 mg at a constant volume of the real sludge suspension of 20 mL (2 wt.% sludge). This parameter is an important fact from an economic point of view, while the optimal amount of nanoadsorbent means that if the active sites are further increased, there is no influence on the removal efficiency, since the available active sites are already fully occupied at a certain metal concentration. In all cases, the removal efficiency increased up to 10 mg MNPs@SiO_2_@GOPTS-Lys (Figure 6). After that, the removal efficiency for all metals starts to continue constantly up to the plateau part, indicating the saturation of the adsorption active sites with metals. While Zn and Cu show a similar adsorption behavior with respect to the amount of nanoadsorbent, the Cr removal efficiency increases progressively for the same amount of adsorbent. This can be attributed to a higher affinity of the active sorption sites to CrO_2_^−4^ ions. From these results, it was concluded that the 10 mg of adsorbent is an optimal value to achieve superior removal efficiency at fixed conditions and was used in the following studies.

#### 3.2.2. Effect of contact time onto simultaneous heavy metal removal from sludge and kinetic modeling

**Figure 7.**
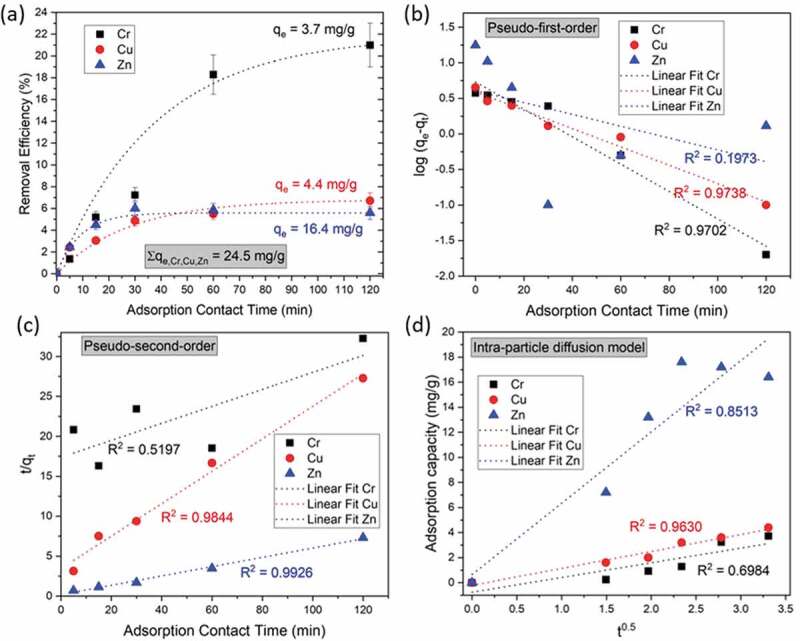
Effect of adsorption contact time on removal efficiency of metals from sludge suspension using MNPs@SiO_2_@GOPTS-Lys at fixed conditions: nanoadsorbent amount 10 mg, pH 10.7, 2 wt% sludge suspension at T = 298 K (a). Fitting of pseudo-first-order (b), pseudo-second-order (c) and intra-particle diffusion model (d) for Cr, Cu and Zn by simultaneous removal from sludge suspension

The influence of contact time on the simultaneous removal of heavy metals from the sludge suspension was investigated in the time range from 5 min to 120 min (Figure 7). The reason for this was that the adsorption operating time is an important parameter that directly influences the economic process efficiency. The efficiency of Zn and Cu removal increased during the first 30 min, then equilibrium with the occupied adsorption active sites was established, and the efficiency of removal was about 6–7% when equilibrium was established, resulting in adsorption capacities of 4.4 and 16.4 mg/g for Cu and Zn, respectively. In contrast, Cr showed a slow surface coverage of MNPs@SiO_2_@GOPTS-Lys until equilibrium was reached after 120 min, resulting in a removal efficiency of about 21% (q_e_ = 3.7 mg/g). From these results, it can be concluded that the equilibrium adsorption time was 120 min and was also used as optimal contact time in other experiments. The total equilibrium adsorption capacity for all metals removed simultaneously was proven to be 24.5 mg/g. Despite the fact that the greater removal efficiency was observed for Cr, the highest adsorption capacity with simultaneous metal removal from the sludge suspension was shown for Zn, which accounted for 16.4 mg/g. It is obvious that Zn and Cu quickly occupy active nanoadsorption sites, while Cr removal increased with extended adsorption time, probably due to the multilayer adsorption until equilibrium was reached. To evaluate the nature of the adsorption mechanism of the simultaneous uptake of Cr, Zn, and Cu from the sludge suspension, three widely used kinetic models, i.e. pseudo-first-order (Figure 7(b)), pseudo-second-order (Figure 7(c)) and intra-particle diffusion models (Figure 7(d)) were investigated [21,61]. The plotted linearized adsorption kinetics curves showed a better correlation of Zn and Cu with the pseudo-second-order, which predicts that the rate-limiting step is chemical adsorption [20] (Figure 7(c)) with correlation factors R^2^ = 0.9844 for Cu and R^2^ = 0.9926 for Zn. In this case, it appears that this model is a dominant mechanism for Zn. In contrast, in the case of Cu, the correlation coefficient for the intra-particle diffusion model was above 0.9 (R^2^ = 0.9630), indicating that the kinetic mechanism can also be partially described on particle diffusion, which partially controls adsorption. For Cr, the overall kinetics are determined by the pseudo-first model (R^2^ = 0.97023), which implies that physical adsorption is a rate-limiting step [20,21,61]. From the results, it could be concluded that in the simultaneous removal of different metals, each metal adsorbs onto MNPs@SiO_2_@GOPTS-Lys at a certain metal concentration in the sludge suspension with a different sorption mechanism.

#### 3.2.3. Influence of native alkaline pH of sludge suspension on heavy metal removal

During the adsorption process, the pH value of the medium (in our case a complex sludge suspension) remains a basic control parameter that influences not only the protonation/deprotonation and surface chemistry of the nanoadsorbent, but also the chemistry of the metal ion species in the medium. In general, sludge from municipal wastewater treatment is subjected to sludge stabilization (e.g. alkaline stabilization, anaerobic digestion, aerobic digestion, and composting) to remove pathogens, pollutants, and odor [46]. Our real sludge suspension sample was treated with alkaline stabilization, which resulted in a very high pH value to minimize organic activity. In fact, the most common composition of non-centrifuged raw sludge is dry matter containing approximately 1% inorganic material and 4% organic material in the form of living organisms such as bacteria, moulds, and carbon-based molecules [4]. Consequently, our true sludge suspension had a very alkaline pH of 10.7. It is known from the literature that metal ions begin to form at a pH of 6 metal hydroxide precipitates [62], indicating that the metals we have selected are essentially in the form of precipitates, although some species are still present in the form of hydrated aqueous ions and are therefore available for adsorption to nanoadsorbent at a pH of the sludge origin. In fact, for Cu, the most important copper species are Cu(OH)_2_ (aq) (this could be due to the fact that Cu(OH)_2_ precipitates are soluble in concentrated alkalis) and some CuOH^+^, also Cu(OH)^−^_3_ complexes were formed at higher pH values [63]. For Zn, the Zn(OH)_2_ precipitate starts to dissolve at pH >8.68 as Zn(OH)^−^_3_ and Zn(OH)_4_^2-^. This could be due to the fact that Zn(OH)_2_ has amphoteric properties, as it reacts with hydroxyl at high pH [63,64]. As far as the pH value of Cr is concerned, it is mainly present as an anion in CrO_4_^2-^ in the sludge in an alkaline environment [65]. However, these data are taken into account for the distribution of pure metal ions in a pure aqueous environment, whereas the sludge suspension is a complex system in which the presence of other cations/anion types is expected, which can strongly influence the metal chemistry. In addition, the presence of organic matter in the sludge suspension results in some metals to be accumulated there and not being available for the adsorption process [6]. Therefore, the complete removal of heavy metals from the sludge is not to be expected, but can only lead to a considerable reduction. This problem could be solved by acid treatment of the sludge suspension by elution of all metals present, but it is known that iron oxide-based NPs dissolve in an acidic environment and a process in this way is also economically unattractive [27]. It must be pointed out that our aim was to minimize the additional changes and to carry out the adsorption tests with the sludge suspension as it is, without any changes being necessary. Similarly, the results from the ZP behavior of MNPs@SiO_2_@GOPTS-Lys (Figure 4(a)) show a negative ZP potential with net value of about – 40 mV at pH around 10–11. This corresponds to deprotonated amino and carboxylic acid groups from the derived lysine backbone on the nanoadsorbent as well as from negatively charged silanol groups. The adsorption efficiency (Table 1) of the selected heavy metal, simultaneously removed from complex sludge suspensions, correlates well with the above-mentioned facts. In native sludge pH, both the metal ion species and the nanoadsorbents are negatively charged, so that the electrostatic repulsion among them can significantly hinder the adsorption process. This is visible, with not very promising removal efficiency being 6%, 7%, and 22% for Cu, Zn, and Cr, respectively (Table 1).

**Table 1.** Results of metal removal from sludge suspension at pH 10.7, 2 wt.% sludge suspension, 10 mg of nanoadsorbent, T = 298 K, contact time 2 h

Nanoadsorbent	Metal	Sludge sample [mg/kg of dried matter]	Treated sludge sample [mg/kg of dried matter]	Removal efficiency [%]	Adsorption capacity (mg metal/g nanoadsorbent)	Sewage Sludge Directive for use in agriculture 86/278/EEC* [mg/kg of dried matter]	Official Gazette the Republic of Slovenia, No. 99/13 in 56/15	
							1. quality class of compost [mg/kg of dried matter]	2. quality class of compost [mg/kg of dried matter]
MNPs@SiO_2_@GOPTS-Lys	Zn	733	687	6	17.2	2,500–1,200	400	1800
	Cu	164	151	7	4.8	1,000–1,750	100	500
	Cr	44.3	34.4	22	3.9	/	100	500
MNPs	Zn	733	711	3	8.8	2,500–1,200	400	1800
	Cu	164	157	4	2.8	1,000–1,750	100	500
	Cr	44.3	42.5	4	0.7	/	100	500

*reference [10]

However, the adsorption capacity (Table 1) showed that this corresponds to a total of 25.9 mg Zn, Cu, and Cr per 1 g MNPs@SiO_2_@GOPTS-Lys when these three metals are removed simultaneously under given conditions. For comparison, the adsorption capacities for bare MNPs were 53% lower compared to MNP@SiO_2_@GOPTS-Lys when these metals were also removed simultaneously under given conditions (Table 1). In addition, the constructed nanoadsorbent shows a high removal efficiency for Cr metal, the most problematic and toxic metal from an environmental point of view [6], while Zn is seen to have the highest adsorption capacity and simultaneously the highest affinity to the nanoadsorbent. If the removal capacity of the nanoadsorbent from real complex multi-metallic sludge systems has been compared with other adsorbents currently available, which are mainly used for the removal of one metal in neutral aqueous model solution, we can conclude that the adsorbent we have developed has a great capacity for the removal of heavy metals (see Table S1 in SM).

All three heavy metals are before and after their removal with magnetic nanoadsorbents still within the limits according to the Sewage Sludge Directive (use of sludge in agriculture) and fall in Slovenia’s second quality class of Official Gazette for the use of sewage sludge as compost for soil improvement (Table 1). Nevertheless, Zn and Cu in the sludge are also approaching the limit values that such sludge could be used as first quality class compost. The latter can be achieved by additional removal steps with successfully regenerated nanoadsorbent. However, when the sludge is used in agriculture, it can be an additional problem that these toxic metals can still accumulate in plants (biosystems) and cause health problems for animals and humans even at trace levels when leaching from the soil into the groundwater [11]. Despite the fact that Zn is a basic component of various metallo-enzymes and an essential micronutrient, its excessive amount can impair reproduction and growth [66]. Similarly, Cu is a component of many enzymes, but its excessive intake can cause serious toxicological concerns, such as nausea, diarrhea, and, in the worst case, death [66,67]. Cr is known to be highly toxic in its hexavalent form and moderately toxic in its trivalent form, most likely due to its strong oxidation properties. Human exposure to Cr compounds has been shown to cause liver and kidney damage, a higher incidence of respiratory cancer and premature death of blood cells [66]. For these reasons, the reduction of the content of the above-mentioned heavy metals in sludge is of great concern also due to their trace toxicity and their accumulation in biosystems.

#### 3.2.4. Regeneration studies of the nanoadsorbent for simultaneous metal removal from sludge suspension

**Figure 8.**
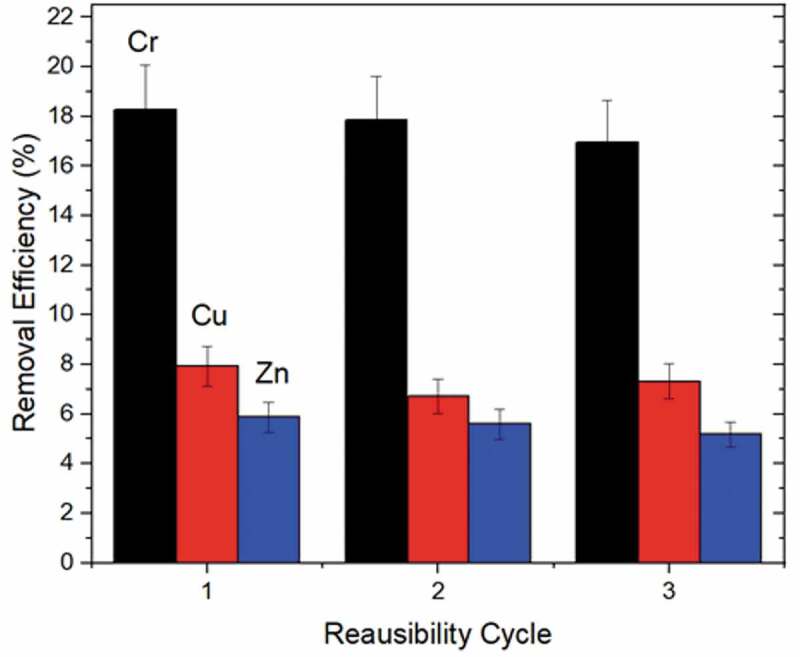
Reusability cycles of MNPs@SiO_2_@GOPTS-Lys after metal removal from sludge suspension using EDTA as an eluent

Reusability cycles are an important parameter for the reuse of adsorbents, since the same adsorbent can be reused for the next adsorption process after the adsorption-desorption process, which significantly influences the economic feasibility of the adsorbent and at the same time contributes to a green and zero-waste concept with waste minimization. Figure 8 shows the efficiency of heavy metal removal from sludge suspensions after the 1^st^, 2^nd^ and 3^rd^ reuse cycle. The removal efficiency with simultaneous uptake of Cr, Cu and Zn metals did not differ significantly, and after the 3^rd^ cycle the removal efficiency is close to the values obtained after the 1^st^ cycle. In addition, the efficient recovery of the nanoadsorbent was verified by infrared spectroscopy of regenerated MNPs@SiO_2_@GOPTS-Lys, and no specific changes in the presence or position of the functional groups of the nanoadsorbent were detected (see Figure S3 in SM). The results suggest that the constructed nanoadsorbent MNPs@SiO_2_@GOPTS-Lys can be used several times for repeated heavy metal removal of different metals without losing its high efficiency together with possible multiple reuse cycles which present economic and environmental advantages of process.

### 3.3. Chemical stability of prepared functional nanostructures in environmental system

**Figure 9.**
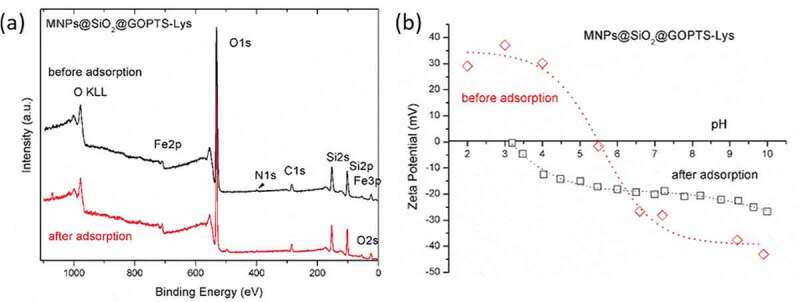
Survey spectra of MNPs@SiO_2_@GOPTS-Lys before and after adsorption from sludge suspension (a) together with the electrokinetic properties of the same nanoadsorbent before and after removal from sludge suspension (b)

The chemical stability of the designed nanoadsorbent was determined with the surface technology XPS before and after the adsorption process from a complex sludge suspension under experimentally optimal conditions, which were previously determined (in 2 wt.% sludge suspension, T = 298 K, 10 mg nanoadsorbent, contact time 2 h, pH 10.7), in order to verify whether after the adsorption of metals from the sludge suspension a change in surface composition occurred which would directly indicate a derived lysine desorption from the surface of the nanoadsorbent, and the latter is not desirable as it contributes to lower adsorption efficiency. The survey spectra of MNPs@SiO_2_@GOPTS-Lys before and after adsorption are shown in Figure 9(a), while the corresponding atomic surface composition is shown in Table 2. Survey spectra of GOPTS-Lys-modified MNPs@SiO_2_ show the elements at binding energies that correspond to the composition of the nanoadsorbent surface. The Fe originates from the maghemite magnetic core, while the Si indicates the presence of the silica layer together with the presence of O. The presence of N is an indicator of the existence of derived lysine, which has also been detected and clearly shows its binding on the MNPs@SiO_2_ surface. In addition, the presence of O and C is also partly due to the derived lysine. The corresponding high-resolution Fe 2p spectra (Figure S4a in SM) of MNPs@SiO_2_@GOPTS-Lys before adsorption show an intense peak at the binding energy of 710.8 eV next to a characteristic satellite, confirming the Fe_2_O_3_ structure [68]. The latter is in high agreement with the XRD results (Figure 1). The Si 2p peak is normally located at an energy of 103.5 eV, which corresponds to the binding of silicon to SiO_2_ [69]. After surface modification of MNPs@SiO_2_ with GOPTS-Lys, the Si 2p peak was shifted to the more negative binding energies (~102.9 eV; see Figure S4b in SM), indicating a silanization process of GOPTS-Lys to MNPs@SiO_2_ and supporting our proposed chemical linkage (Scheme 1). The high-resolution spectra for N 1s (Figure S4c in SM) indicate the presence of N in NH_2_ form [70]. From the C 1s spectrum, the peak at about 288.9 eV can also be identified (Figure 4Sd in SM), typical for O–C=O groups at nanoadsorbent [69].

**Table 2.** Elemental surface composition of MNPs@SiO_2_@GOPTS-Lys before and after the adsorption from the sludge suspension

Sample/at.%	C	N	O	Si	Fe	Na
MNPs@SiO_2_@GOPTS-Lys, before adsorption	8.0	0.7	66.9	23.6	1.0	-
MNPs@SiO_2_@GOPTS-Lys, after adsorption	7.9	0.6	65.4	23.2	1.3	1.5

Figure 9(a) and Table 2 show that the atomic surface composition did not change significantly before and after the adsorption process. Although we have not detected the heavy metals after adsorption from the sewage sludge, this does not mean that they are not present on the adsorbent surface, but it may be that their surface concentration is below the detection limit of XPS surface technique (which is typically ~0.5 at.%). In addition, the atomic surface composition of N is almost constant before and after adsorption, which shows that the derived amino acid is strongly covalently bound to MNPs@SiO_2_. From this, it can be concluded that our proposed chemical coupling mechanism has indeed been confirmed and shows a great potential of the lysine-based nanoadsorbent for use in the removal of heavy metals from sludge. It is well known that the poorly and not strongly bound nanoadsorbent can cause ligand desorption, which may be directly related to reduced efficiency of heavy metal removal.

In order to reveal the influence of the adsorption process of heavy metal on the electrokinetic behavior of the nanoadsorbents, the ZP curves as a function of the pH value before and after sorption from sludge suspension were carried out (Figure 9(b)). The ZP results of the metal-adsorbed nanosorbent show a shift of the IEP from pH 5.5 to pH 3 with smaller negative absolute values of ZP in comparison to MNPs@SiO_2_ (Figure 4(a)) after the cleaning process. This may be caused by the adsorbed heavy metals from sludge suspensions to amino groups (in some extend probably also to carboxyl and some negatively charged silanol groups), which do not seem to be more accessible for protonation due to the formation of amino-metal complexes, and according to the activity series of the metals in aqueous solution, many of them have a high affinity to amino groups (e.g. Cu) [71,72]. The latter could indicate that the heavy metals may be firmly bound in the shear plane, as explained by the DLVO theory [28] and proved by upper shifting of negative zeta potential plateau value (Figure 9(b)). Indeed, a more acidic character of the adsorbed heavy metals may be correlated with the presence of various metals known to act as acid [73].

## 4. Conclusions

We report on the chemical linkage of the epoxy-organosilane with the amino acid lysine and its functionalization on silica-coated MNPs. The synthesized nanostructured were used as adsorbents for the simultaneous removal of heavy metals from the real environmental system, from complex sludge suspensions. The nanoscale and core-shell structure of MNPs@SiO_2_@GOPTS-Lys was revealed by TEM analysis, while the EDS analysis also supported the presence of all elements except N, which was well below of detection limit. The presence of specific functional groups with different modification steps was confirmed by ATR-FTIR spectroscopy and electrokinetic measurements. Mass loss measurements showed that the silica-coated MNPs surface was partially covered with GOPTS-Lys, which amounted to approximately 1 molecule of GOPTS-Lys per nm^2^. Remarkably, the large magnetic response of the nanoadsorbent ensured its easy and fast removal after a complicated adsorption process. The experimental results in the simultaneous removal of the most critical heavy metals in the sludge suspension (i.e. Zn, Cu, Cr) showed that the alkaline pH has the major influence on metal adsorption, whereas with native sludge pH, both the metal ion species and the nanoadsorbents are negatively charged so that the electrostatic repulsion among them can significantly impede the adsorption process. In fact, the adsorption capacity shows a large amount of metal per mg of nanoadsorbent with respect to bare MNPs and a high efficiency in removing Cr. When metals were removed simultaneously as a function of time, a different behavior for metals was observed and explained by proposed adsorption kinetic mechanisms. For Zn and Cu, the dominant adsorption mechanism is chemical adsorption, whereas for Cr, physical adsorption is a rate-limiting step. Importantly, the removal efficiency of the nanoadsorbent did not change during the reuse cycles, indicating the feasibility of repeated heavy metal removal from the sludge by the nanoadsorbent and its environmental and economic benefits. The most important practical factor, i.e. the chemical stability of the nanoadsorbent (i.e. possible-derived lysine desorption), was checked with the surface-sensitive technique XPS, and the comparison of the elemental surface composition of MNPs@SiO_2_@GOPTS-Lys before and after the adsorption process from real complex sludge suspensions did not show significant changes. In addition, the electrokinetic measurement showed changes in the nanoadsorbent IEP and surface charge after metal uptake from sludge suspension possibly caused by metal adsorption. Overall, the developed nanoadsorbent is an innovative, efficient, environmentally friendly and stable sorbent that can be applied for simultaneous metal removal under more complicated environmental conditions and gives the waste (such as sludge) the possibility of recycling.

## Supplementary Material

Supplemental Material
