# Supplementary material for: Magnetic nanostructures functionalized with a derived lysine coating applied to simultaneously remove heavy metal pollutants from environmental systems
Source: Sci Technol Adv Mater. 2021 Jan 22;22(1):55–71. doi: 10.1080/14686996.2020.1865114 (PMC7832508; doi:10.1080/14686996.2020.1865114)
Supplement: Supplemental Material [file TSTA_A_1865114_SM5176.pdf]

Supplemental Material for

Magnetic nanostructures functionalized with a derived lysine coating applied to simultaneously remove heavy metal pollutants from environmental systems

Olivija Plohl[a,*], Marjana Simonič[b], Ken Kolar[a], Sašo Gyergyek[b,c], Lidija Fras Zemljič[a]

[a]University of Maribor, Faculty of Mechanical Engineering, Laboratory for Characterization and Processing of Polymers, Smetanova 17, 2000 Maribor, Slovenia

[b]University of Maribor, Faculty of Chemistry and Chemical Engineering, Smetanova 17, 2000 Maribor, Slovenia

[c]Jožef Stefan Institute, Jamova 39, 1000 Ljubljana, Slovenia

Corresponding Author:

E-mail[*]: olivija.plohl@um.si

Details of chemicals and reagents used:

$FeSO_4$ $7H_2O$, NaOH (> 98%), NH_4OH (25% aqueous solution) and acetone (≥ 99.5%) were obtained from Honeywell (Seelze, Germany). $Fe_2(SO_4)_3$ $7H_2O$, tetraethylorthosilicate (TEOS, ≥98%), HCl (≥ 37%), L-lysine crystallized (≥98%) and 3-glycidoxypropyltrimethoxysilane (GOPTS, 98%) were ordered from Sigma-Aldrich (Taufkirchen, Germany). Citric acid (≥ 99.5%, water free) was purchased from Roth (Karlsruhe, Germany) and absolute EtOH (anhydrous) was obtained from CarloErba (Val de Reuil, France). All reagents were used as received, without additional purification. Ultrapure water (sourced from Milli-Q, Millipore Corporation, Massachusetts, USA, with a resistivity of 18.2 MΩ cm) was used in all experiments.

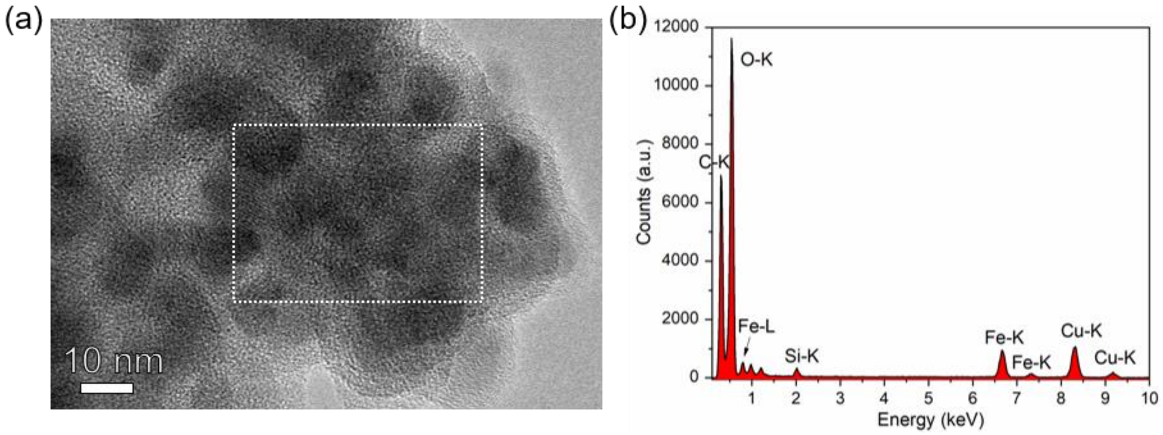

Figure S1: TEM image of MNPs@SiO2@GOPTS-Lys with white square (a) enclosed, indicating where the corresponding EDXS was acquired, and the results of this are shown in (b). The presence of Cu is due to a Cu-supported film.

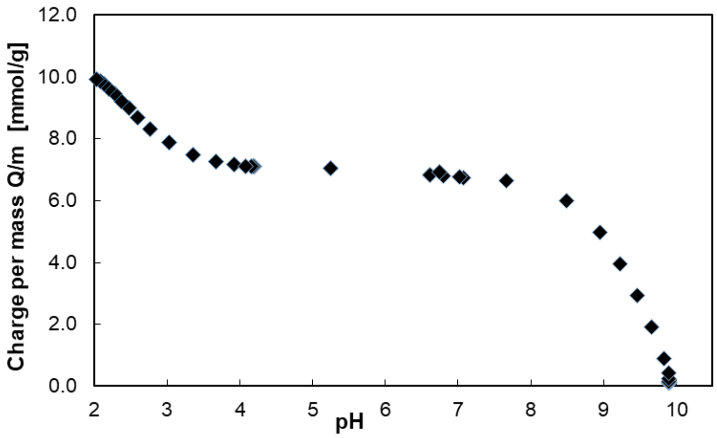

Figure S2: Potentiometric titration curve of derivatised lysine.

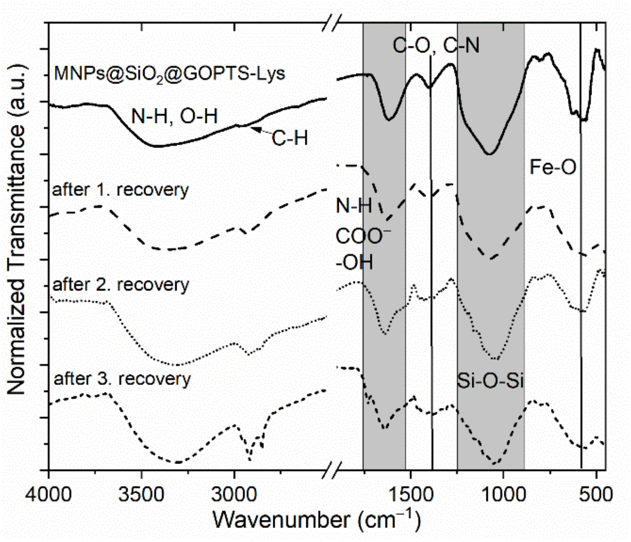

Figure S3: ATR-FTIR spectrum of the initial MNP@SiO_2@GOPTS-Lys and its comparison after different recovery/regeneration steps.

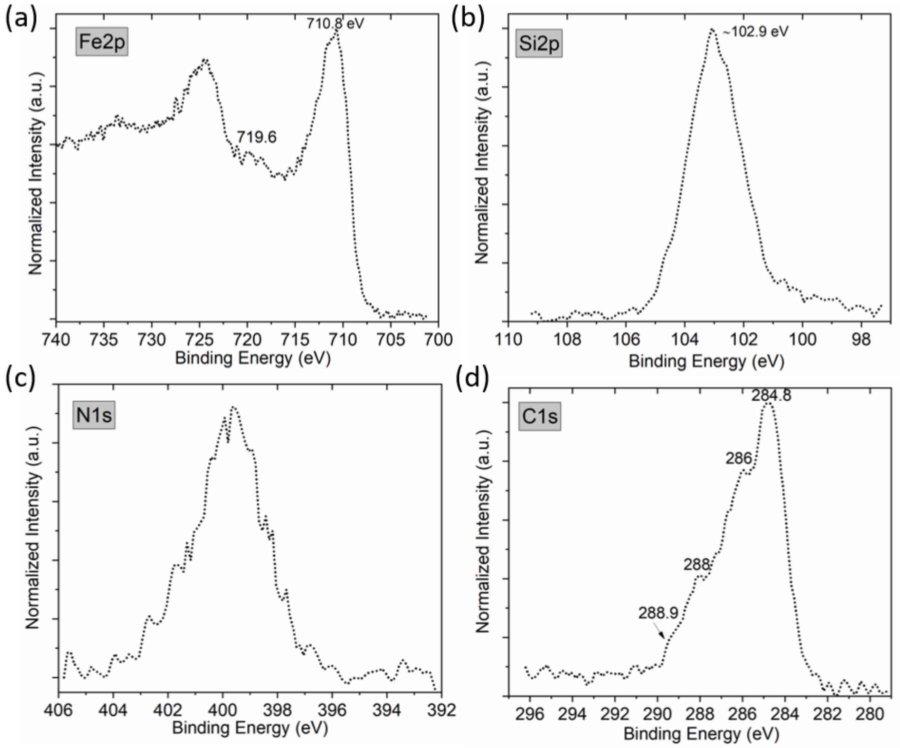

Figure S4: High-resolution spectra of a) Fe 2p b) Si 2p, c) N 1s and d) C 1s of MNPs@SiO_2@GOPTS-Lys.

Table S1. Comparison of MNPs@SiO_2@GOPTS-Lys adsorption capacities to other Zn, Cu and Cr adsorbents removal.

Adsorbent materials	pH of adsorption	Real/model system	Adsorption capacity (mg/g)				References
			Zn	Cu	Cr	Σmetals	
Crosslinked chitosan with epichlorohydrin	7	Model individual metal aqueous system	10.2	35.5	NR	/	[1]
Fe_3O_4/MnO_2	6.3	Model individual metal aqueous system	8.1	9.8	NR	/	[2]
Potato peel charcoal	6	Model individual metal aqueous system	NR	0.39	NR	/	[3]
MWCNTs	9	Model individual metal aqueous system	NR	3.49	NR	/	[4]
Mesoporous magnetic carbon nanocomposite fabrics	7	Model individual metal aqueous system	NR	NR	3.74	/	[5]
Magnetic Graphene nanocomposites decorated with core@double-shell nanoparticles	7	Model individual metal aqueous system	NR	NR	1.03	/	[6]
Xanthate-modified magnetic chitosan	5	Ternary metal system in model aqueous solution	7.6	26.3	NR	62.5	[7]
Micron Fe	3	Heavily contaminated groundwater and	NR	NR	1.33-2.16	/	[8]

		chromium ore processing residue samples					
Micron Fe	3	Heavily contaminated groundwater	NR	NR	1.53-1.75	/	[8]
C-phenylcalix[4]pyrogallolarene	5	Model individual metal aqueous system	NR	8.140	16.86	/	[9]
Magnetic-chitosan nanoparticles	10-11	Simultaneous removal from real sludge suspension	96.2	10.1	NR	134.7	[10]
nZVI-Fe_3O_4	3	Model individual metal aqueous system	NR	NR	100	NR	[11]
Nanoscale Zero-Valent Iron (nZVI) assembled on magnetic Fe_3O_4/graphene	8	Model individual metal aqueous system	NR	NR	66.2	NR	[12]
**MNPs@SiO_2@GOPTS-Lys**	**10.7**	**Simultaneous removal from real sludge suspension**	**17.2**	**4.8**	**3.9**	**24.5**	**This work**

Supplemental References

[1] Chen AH, Liu SC, Chen CY, et al. Comparative adsorption of Cu(II), Zn(II), and Pb(II) ions in aqueous solution on the crosslinked chitosan with epichlorohydrin. J Hazard Mater. 2008;154:184–191.

[2] Kim E-J, Lee C-S, Chang Y-Y, et al. Hierarchically Structured Manganese Oxide-Coated

Magnetic Nanocomposites for the Efficient Removal of Heavy Metal Ions from Aqueous Systems. ACS Appl Mater Interfaces [Internet]. 2013;5:9628–9634. Available from: http://pubs.acs.org/doi/abs/10.1021/am402615m.

[3] Aman T, Kazi AA, Sabri MU, et al. Potato peels as solid waste for the removal of heavy metal copper(II) from waste water/industrial effluent. Colloids Surfaces B Biointerfaces. 2008;63:116–121.

[4] Stafiej A, Pyrzynska K. Adsorption of heavy metal ions with carbon nanotubes. Sep Purif Technol. 2007;58:49–52.

[5] Zhu J, Gu H, Guo J, et al. Mesoporous magnetic carbon nanocomposite fabrics for highly efficient Cr(vi) removal. J Mater Chem A. 2014;2:2256–2265.

[6] Zhu J, Wei S, Gu H, et al. One-pot synthesis of magnetic graphene nanocomposites decorated with core@double-shell nanoparticles for fast chromium removal. Environ Sci Technol. 2012;46:977–985.

[7] Zhu Y, Hu J, Wang J. Competitive adsorption of Pb(II), Cu(II) and Zn(II) onto xanthate-modified magnetic chitosan. J Hazard Mater [Internet]. 2012;221–222:155–161. Available from: http://dx.doi.org/10.1016/j.jhazmat.2012.04.026.

[8] Cao J, Zhang WX. Stabilization of chromium ore processing residue (COPR) with nanoscale iron particles. J Hazard Mater. 2006;132:213–219.

[9] Jumina, Priastomo Y, Setiawan HR, et al. Simultaneous removal of lead(II), chromium(III), and copper(II) heavy metal ions through an adsorption process using C-phenylcalix[4]pyrogallolarene material. J Environ Chem Eng [Internet]. 2020;8:103971.

Available from: https://doi.org/10.1016/j.jece.2020.103971.

[10] Bratina B, Šorgo A, Kramberger J, et al. From municipal/industrial wastewater sludge and FOG to fertilizer: A proposal for economic sustainable sludge management. J Environ Manage. 2016;183:1009–1025.

[11] Lv X, Xu J, Jiang G, et al. Highly active nanoscale zero-valent iron (nZVI)-Fe3O4 nanocomposites for the removal of chromium(VI) from aqueous solutions. J Colloid Interface Sci [Internet]. 2012;369:460–469. Available from: http://dx.doi.org/10.1016/j.jcis.2011.11.049.

[12] Lv X, Xue X, Jiang G, et al. Nanoscale Zero-Valent Iron (nZVI) assembled on magnetic Fe3O4/graphene for Chromium (VI) removal from aqueous solution. J Colloid Interface Sci [Internet]. 2014;417:51–59. Available from: http://dx.doi.org/10.1016/j.jcis.2013.11.044.
